# Atypical responses to faces during binocular rivalry in early glaucoma

**DOI:** 10.3389/fnins.2023.1151278

**Published:** 2023-05-25

**Authors:** Galia Issashar Leibovitzh, Graham E. Trope, Irfan N. Kherani, Yvonne M. Buys, Luminita Tarita-Nistor

**Affiliations:** ^1^Krembil Research Institute, Donald K. Johnson Eye Institute, Toronto Western Hospital, Toronto, ON, Canada; ^2^Department of Ophthalmology and Vision Sciences, University of Toronto, Toronto, ON, Canada

**Keywords:** glaucoma, binocular rivalry, face perception, neurodegeneration, optic neuropathy

## Abstract

**Purpose:**

Glaucoma is a progressive optic neuropathy that damages retinal ganglion cells and a neurodegenerative disease as it affects neural structures throughout the brain. In this study, we examined binocular rivalry responses in patients with early glaucoma in order to probe the function of stimulus-specific cortical areas involved in face perception.

**Methods:**

Participants included 14 individuals (10 females, mean age 65 ± 7 years) with early pre-perimetric glaucoma and 14 age-matched healthy controls (7 females, mean age 59 ± 11 years). The 2 groups were equivalent in visual acuity and stereo-acuity. Three binocular rivalry stimulus pairs were used: (1) real face/house, (2) synthetic face/noise patch, and (3) synthetic face/spiral. For each stimulus pair, the images were matched in size and contrast level; they were viewed dichotically, and presented centrally and eccentrically at 3 degrees in the right (RH) and in the left hemifield (LH), respectively. The outcome measures were rivalry rate (i.e., perceptual switches/min) and time of exclusive dominance of each stimulus.

**Results:**

For the face/house stimulus pair, rivalry rate of the glaucoma group (11 ± 6 switches/min) was significantly lower than that of the control group (15 ± 5 switches/min), but only in the LH location. The face dominated longer than the house in the LH for both groups. Likewise, for the synthetic face/noise patch stimulus pair, rivalry rate of the glaucoma group (11 ± 6 switches/min) was lower than that of the control group (16 ± 7 switches/min) in the LH, but the difference failed to reach significance. Interestingly, the mixed percept dominated less in glaucoma than in the control group. For the synthetic face/spiral stimulus pair, the glaucoma group had lower rivalry rate at all 3 stimulus locations.

**Conclusion:**

This study reveals atypical responses to faces during binocular rivalry in patients with early glaucoma. The results may be suggestive of early neurodegeneration affecting stimulus-specific neural structures involved in face processing starting in the pre-perimetric phase of the disease.

## Introduction

Glaucoma is the leading cause of irreversible blindness in the working age population globally, creating a heavy burden on patients, their families and health systems (Quigley and Broman, 2006; Varma et al., 2011; Tham et al., 2014; Zhang et al., 2021). Due to aging of the population and increased life expectancy, it is estimated that 112 million people will be affected by glaucoma in 20 years (Tham et al., 2014).

Glaucoma is a progressive optic neuropathy that results in damage and death of retinal ganglion cells (RGCs) with elevated intraocular pressure being a major risk factor but not necessarily the cause of optic nerve head injury and RGC loss. A large proportion of people with ocular hypertension do not develop glaucoma, and for those who do develop the disease, lowering the eye pressure only results in slowing of the progression, and not curing the disease (Kass et al., 2002, 2021). In addition, glaucoma can develop in the absence of high intraocular pressure, suggesting that maybe glaucoma represents a group of ocular pathologies with different pathogenesis, although the resulting visual impairment is strikingly similar for all. A growing body of research in the past few decades suggests that glaucoma is also a neurodegenerative disease because it affects neural structures throughout the visual system as well as in more distal areas. Post-mortem histopathological examination of the brain of a patient with advanced glaucoma showed degeneration in the intracranial optic nerve, lateral geniculate nuclei, and visual cortex (Gupta et al., 2006). With the advancement in brain imaging techniques, structural changes have been detected in the primary visual pathway including the optic nerve, optic chiasm, optic tract, lateral geniculate nuclei, optic radiations, and the visual cortex of occipital lobes (Garaci et al., 2009; Hernowo et al., 2011; Zhang et al., 2012; Frezzotti et al., 2014; Carvalho et al., 2021, 2022). These changes are associated with glaucoma severity (Garaci et al., 2009; Zhang et al., 2012; Frezzotti et al., 2014; Tellouck et al., 2016) and may be explained by anterograde trans-synaptic axonal degeneration (Gupta and Yücel, 2007). However, the degeneration is not limited to the primary visual pathway, as changes in the cortex and the white matter have been found throughout the brain. These areas include (but are not limited to) superior frontal gyrus, inferior frontal gyrus, frontoparietal cortex, precuneus, lingual gyrus, hippocampi, right inferior occipital gyrus, right inferior temporal gyrus, inferior longitudinal fasciculus, right occipital lobe white matter, superior longitudinal fascicle, and corpus callosum (Chen et al., 2013; Williams et al., 2013; Frezzotti et al., 2014; Boucard et al., 2016; Nucci et al., 2020). These changes support the idea that other degenerative mechanism likely exists in glaucoma.

It is not understood if changes observed throughout the glaucomatous brain manifest in functional changes. Recently, we employed a binocular rivalry paradigm to probe the function of specific brain structures in patients with early glaucoma (Tarita-Nistor et al., 2019, 2020; Issashar Leibovitzh et al., 2022). Binocular rivalry occurs when 2 different images are presented separately to each eye at the same retinal location and are viewed dichotically. In such cases, the healthy visual system cannot integrate the 2 images into a stable, unified percept. Rather, these images compete for perceptual awareness: as one image is perceived, it is supressed by the other image moments later, in a continuous cycle. The perceptual dominance dynamics during binocular rivalry involve reciprocal inhibitory interactions between populations of neurons as well as neural adaptation of the activity associated with the dominant stimulus at any given time. The excitatory and inhibitory processes of populations of neurons during binocular rivalry may be regulated by the glutamate—gamma aminobutyric acid (GABA) neurotransmitter balance, but the exact mechanism of this phenomenon has not been entirely elucidated. Wilson (2003) suggested that binocular rivalry may involve 2 stages of processing, depending on stimulus complexity. Rivalry processes of complex stimuli may involve different mechanisms as opposed to simple stimuli which are handled earlier in the visual system. In experiments of binocular rivalry using simple stimuli (i.e., sinewave gratings), compared to healthy controls, patients with early glaucoma had lower rivalry rate (i.e., number of perceptual switches per minute) and faster dominance wave propagation in conditions involving interhemispheric transfer (Tarita-Nistor et al., 2019, 2020; João et al., 2021). Also, perceptual grouping (of spatially separated identical stimuli) during binocular rivalry appears to be slower in patients with early glaucoma, regardless of the stimulus location presentation (Issashar Leibovitzh et al., 2022). This evidence points to a dysregulation of inter-hemispheric transfer involving corpus callosum and likely a weakening in the strength of neural connectivity of the visual cortex involved in early visual processing.

To date, the only stimuli used in binocular rivalry experiments for patients with glaucoma have been sinewave gratings of a specific spatial frequency. These stimuli lack complexity, and likely involve the first stage of binocular rivalry processing. More complex rivalry stimuli may reveal changes in more distal areas of the extrastriate cortex. Some stimuli are processed in stimulus specific areas; for example, faces are processed partially in a neural network (i.e., core system) composed of occipital face area (OFA), fusiform face area (FFA), and posterior superior temporal sulcus (pSTS), while houses/objects are processed in the parahippocampal place area (PPA) (Tong et al., 1998; Haxby et al., 2000). In a landmark study, using functional magnetic resonance imaging (fMRI) to monitor responses during binocular rivalry using a house and a face as stimuli, Tong et al. (1998) reported that visual perception of a face during binocular rivalry matched the FFA activation while visual perception of the house matched the PPA activation. A replay condition in which the rivalry responses were played back to match the perceptual switching during rivalry produced the same neural activation, suggesting that the rivalry was already resolved by the time the visual information reached these extrastriate areas. Currently there is no clear understanding of where exactly rivalry of the higher order stimuli is resolved, but, as Wilson's model suggests, these rivalry processes are different from those involving simple stimuli (Wilson, 2003).

In this study, we examined binocular rivalry responses in patients with pre-perimetric glaucoma (no or minimal visual field defects), in order to probe the function of stimulus-specific cortical areas involved in face perception. We used 3 stimulus pairs of different complexity; in each pair one stimulus was always a face, while the other stimulus was a different visual object (i.e., house, noise patch, and a spiral). The face stimuli were neutral in emotions and static, and therefore only the FFA (concerned with face recognition) and OFA (implicated in early representation of faces) in the core system were likely involved in the face processing. There is a strong right hemispheric lateralization for the network involved in face perception, but not for other object-selective cortical areas, and therefore we examined rivalry responses when the stimuli were presented centrally, and in each hemifield (i.e., left and right) (Yovel et al., 2008; Frässle et al., 2016). Based on our previous findings and on the fact that imaging studies show changes throughout the glaucomatous brain, we hypothesized that patients with glaucoma will continue to show deficits in rivalry responses involving higher order visual stimuli.

## Materials and methods

### Participants

Fourteen individuals (10 female, mean age 65 ± 7 years) with pre-perimetric glaucoma and 14 age-matched healthy controls (7 female, mean age 59 ± 11 years) were included in this study. Patients were recruited from the glaucoma service at the Toronto Western Hospital, University Health Network, Toronto, Canada. They were diagnosed by an experienced glaucoma specialist (GET) based on clinical examination, family history, visual field mean deviation (MD), and observed deterioration in consecutive optical coherence tomography (OCT) imaging results. Two important criteria were required to diagnose pre-perimetric glaucoma: (1) the presence of glaucomatous changes in the optic disc appearance that were also associated with decreased thickness of the retinal nerve fiber layer (RNFL) detected by spectral domain OCT, and (2) no evidence of significant visual field defects detected with standard automated perimetry (Lisboa et al., 2012; Shiga et al., 2018). Only patients with pre-perimetric open angle glaucoma with no or minimal visual field defects, normal visual acuity (i.e., 20/25 or better) and stereopsis were included. All patients had their intraocular pressure normalized with topical treatment. In addition, they had no significant interocular asymmetry in clinical measures, including in visual acuity (*p* = 0.4), visual field sensitivity (*p* = 0.3), retinal nerve fiber layer thickness (RNFL) (*p* = 0.4), average (*p* = 0.7) and vertical (*p* = 0.6) cup-to-disc ratios, and intraocular pressure (*p* = 0.3). Control participants were volunteers with no ocular history, normal visual acuity and stereopsis. The control participants confirmed verbally that they had had an ophthalmic examination within 2 years, there were no pathological findings or suspicion of any eye diseases, and their habitual correction was updated. Exclusion criteria included comorbid ocular pathologies (except for mild cataract), diabetes and a history of neurological diseases or cognitive impairment. The demographic and clinical characteristics of the glaucoma and control group are presented in Table 1. This study was approved by the University Health Network Research Ethics Board and conducted in accordance with the tenets of the declaration of Helsinki. Written informed consent was obtained from all participants. The testing of all participants was made in the Ocular Motor Laboratory at the Toronto Western Hospital.

**Table 1:** Demographic and clinical characteristics for the glaucoma and control group, along with the p values for the independent-sample t-tests showing the differences between the 2 groups.

	**Glaucoma**	**Control**	***p*-value**
N [F/M]	14 [10/4]	14 [7/7]	–
Age (years)	65 ± 7	59 ± 11	0.07
Stereo acuity (arc sec)	16 ± 3	16 ± 4	0.83
**Visual acuity (logMAR)**			
Binocular	−0.04 ± 0.08	−0.09 ± 0.10	0.17
Right eye	0.04 ± 0.09	−0.03 ± 0.06	0.03
Left eye	0.02 ± 0.09	−0.03 ± 0.05	0.09
**Visual field mean deviation (dB)**			
Right eye	0.47 ± 1.35	–	–
Left eye	0.18 ± 1.13	–	–
**Retinal nerve fiber layer (**μ**m)**			
Right eye	86 ± 11	–	–
Left eye	84 ± 10	–	–
**Average cup-to-disc ratio**			
Right eye	0.66 ± 0.16	–	–
Left eye	0.66 ± 0.15	–	–
**Vertical cup-to-disc ratio**			
Right eye	0.63 ± 0.15	–	–
Left eye	0.64 ± 0.12	–	–
**Intraocular pressure (mmHg)**			
Right eye	13.0 ± 2.5	–	–
Left eye	12.4 ± 3.5	–	–

### Apparatus

Clinical measures were functional and structural. The functional measures consisted of monocular and binocular visual acuity, stereoacuity and visual field sensitivity, while structural measures were RNFL thickness and average and vertical cup-to-disc ratios. Visual acuity was measured at 6 m with a computerized version of the Early Treatment Diabetic Retinopathy Study chart (single line) using the Accommodata Stimuli System, Version 3.5 (Haag–Streit, Mason, OH). During testing, participants wore their habitual corrective spectacles; a letter-by-letter scoring system was used and visual acuity values were reported in logMAR units. Stereoacuity was measured using the Random Dot Stereoacuity Test (Good-Lite Company, Elgin, IL). Visual field sensitivity was obtained for each eye with the Humphrey Field Analyzer (model HFA-II 750; Carl Zeiss Meditec, Dublin, CA) using the monocular 24–2 Swedish Interactive Threshold Algorithm-Standard. Visual field mean deviation was recorded in dB. In addition, spectral domain optical coherence tomography scans (OCT, Cirrus; Carl Zeiss Meditec, Dublin, CA) were performed for each eye using a 200 × 200 optic disc cube protocol, and peripapillary RNFL thickness, average and vertical cup-to-disc ratio were recorded. The visual field tests and OCT scans were not obtained for the healthy controls due to safety concerns for COVID-19 exposure and restricted access to the instruments for patients only.

**Figure 1:**
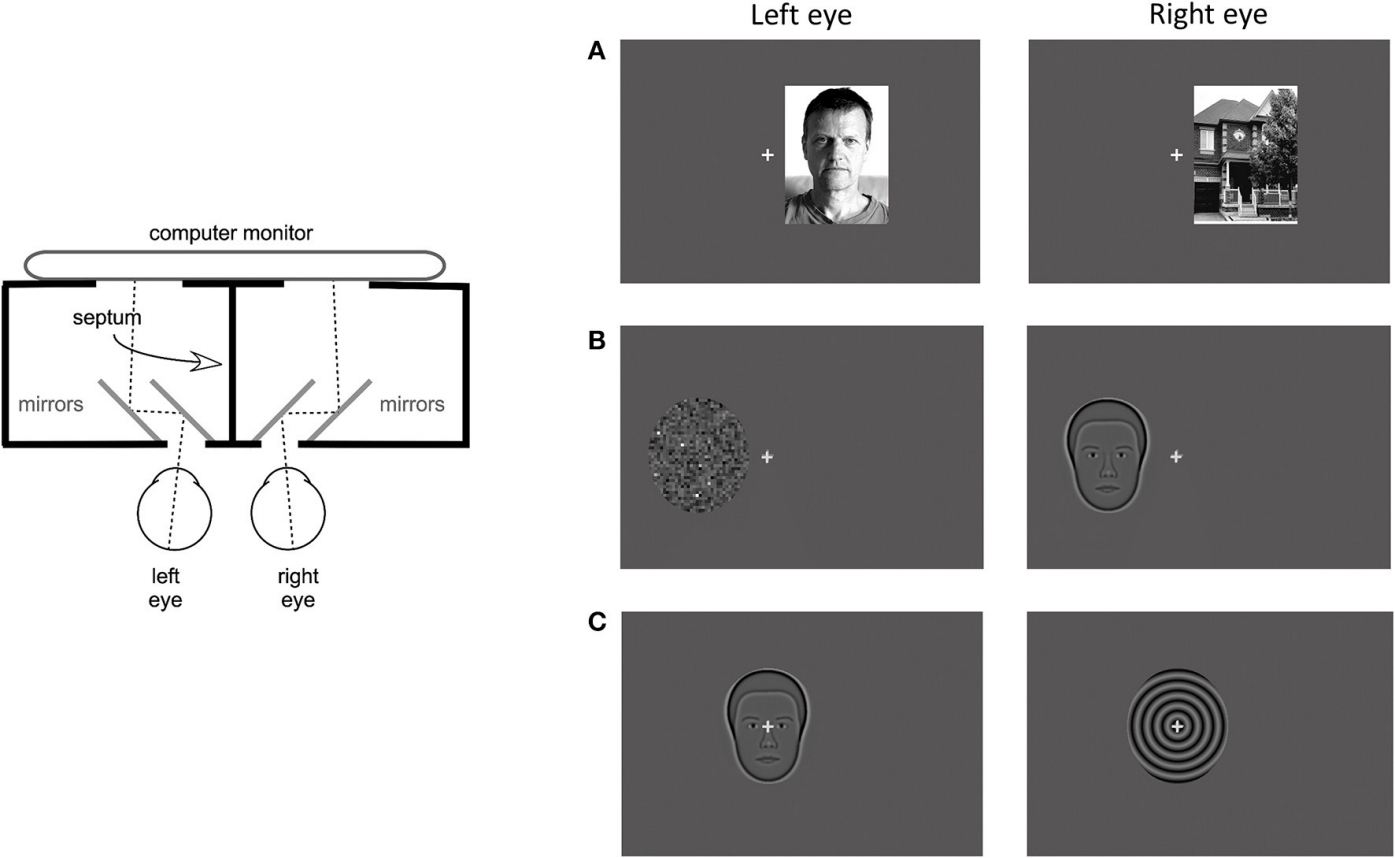
Apparatus and stimuli for the rivalry experiment. **(Left)** Panel shows a diagram of the apparatus that included a double-mirror stereoscope and the septum which ensured dichoptic presentation of the stimuli. **(Right)** Panel shows examples of the 3 stimulus pairs: **(A)** coherent complex global stimuli (face/house), **(B)** mixed stimuli, one global with reduced complexity and one simple with no spatial structure (synthetic face/noise patch), and **(C)** coherent global stimuli with reduced complexity (synthetic face/spiral). Each set was presented at 3 locations: centrally, and eccentrically in the left and right hemifields.

Psychophysical measures were recorded during rivalry test and consisted of rivalry rate and total time of exclusive percept dominance. The rivalry test setup used was the same as previously reported (Tarita-Nistor et al., 2020; Issashar Leibovitzh et al., 2022). In summary, the rivalry stimuli were created using a graphics and psychophysics software VPixx (VPixx Technologies, Inc., Montreal, QC) and projected on an iMac computer screen with a resolution of 1,680 × 1,050 pixels. Participants viewed the stimuli through a double-mirror stereoscope placed in front of the computer on an adjustable table. The stimulus area on the monitor was surrounded by a black mask, and a vertical septum ensured that different stimuli were seen by the left and the right eyes separately (Figure 1, left panel).

**Figure 2:**
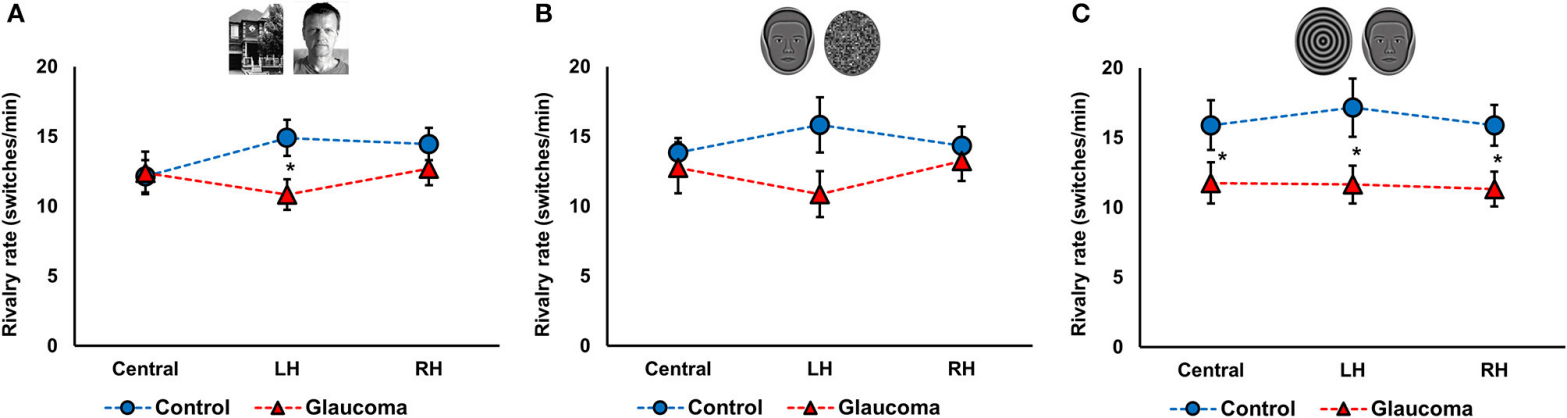
Rivalry rate at three locations for the control and glaucoma groups, for the three stimulus pairs. **(A)** Shows the results for the face/house pair, **(B)** for the synthetic face/noise patch pair, and **(C)** for synthetic face/spiral pair. Error bars are ±1 SE. The star sign * shows significant differences between the 2 groups, corrected for multiple comparisons.

Binocular rivalry stimuli were presented at 3 locations: centrally, 3 degrees eccentrically on the horizontal direction in the left hemifield (LH), and in the right hemifield (RH). They were shown on a dark gray background with luminance of 16 cd/m^2^. A 0.5 degree gray fixation cross was always presented centrally, in all conditions. The eccentricity was measured from the fixation cross to the middle of the stimulus. In this experiment, 3 stimulus pairs were used, each pair including a face. The first stimulus pair consisted of complex global stimuli of a real face with neutral emotions and a house (face/house pair, Figure 1A). These stimuli are broadband in spatial frequency and orientation and both had 80% contrast. Their shape was rectangular with a size of 4.5 degrees width and 6 degree height. The second stimulus pair consisted of simpler stimuli of the average synthetic male face and a noise patch with a granularity of 5 pixels or 0.129 degrees (synthetic face/noise patch pair, Figure 1B). The average synthetic male face (described in McCulloch et al., 2011) was downloaded from the Freiburg Vision Test at https://michaelbach.de/fract/download.html. While the average synthetic face is a coherent global stimulus, albeit not as complex as a real face, the noise patch lacks the coherent spatial structure, rendering it a simple stimulus with no global features. These stimuli had an average contrast of 17% and their shape was an oval with a size of 4.4 degree width and 5 degree height. The third stimulus pair consisted of global stimuli with reduced complexity: the average synthetic male face (described above) and a spiral with a spatial frequency of 2 cycles per degree (synthetic face/spiral pair, Figure 2C). These stimuli had the same shape, size and contrast as the second stimulus pair.

Rivalry responses were recorded using a button-response box connected to a PC computer and an in-house software written in Visual Basic (Microsoft, Albuquerque, NM, US). The participants pressed and held the left button for the face percept, the right button for the other visual stimuli (i.e., house, noise patch, or spiral), and both buttons simultaneously for the mixed percept. The software recorded rivalry rate and the total time of percept dominance (i.e., how long stimulus or piecemeal percepts were reported during one trial).

### Procedure

All clinical and psychophysical measures were obtained in a single session. Monocular and binocular visual acuity, stereoacuity, and binocular rivalry tests were administered in the laboratory for all participants. The visual field tests and the OCT scans were performed on the same day as part of standard of care for the glaucoma cohort.

For the binocular rivalry test, participants were seated in an adjustable chair and had their head stabilized with a chin rest. A double mirror stereoscope was placed in front of them on an adjustable table, along with the computer screen where the stimuli were projected as shown in Figure 1, left panel. The participant's eyes and the center of the screen were adjusted to be at the same level. All participants confirmed that they saw only one fixation cross when looking through the double mirror stereoscope. The participants held the button-response box with both hands and were asked to press and hold the left button if they perceived a face, the right button if they perceived other visual stimuli (i.e., house, noise patch, spiral), or both buttons if they perceived a mixed image. They were instructed to hold the button pressed for as long as they perceived one stimulus, then switch to the other when the percept changed, or hold both buttons pressed for as long as they saw a mixed percept. A practice trail using stimuli presented in the central location was run to ensure that participants understood the task. The practice trial and the experiment were conducted in a darkened room where the only light source was the computer monitor on which the stimuli were projected. Binocular rivalry was tested in 9 conditions: 3 locations (central, LH, RH) × 3 stimulus pairs (face/house, synthetic face/noise patch, synthetic face/spiral). Each condition was 60 s long and they were presented randomly, with short breaks in between. The timing of each condition started with the first press of the button-response box, when the participants felt they were ready to begin. For all the LH and RH conditions, the participants were repeatedly reminded to keep their eyes steady on the fixation cross, but to pay attention to the eccentric rivalrous stimuli. The face stimulus was presented randomly to the left or to the right eye in the 9 conditions.

### Data analysis

The main outcome measures were rivalry rate and total time of exclusive dominance of each percept. Rivalry rate was reported as the number of perceptual switches per minute. The total time of percept dominance was defined as the cumulative time of exclusive dominance of each percept during a trial.

Data were analyzed primarily with mixed factorial analyses of variance (ANOVAs). For each stimulus pair, rivalry rate was analyzed with a 3 (Location: central, LH, RH) × 2 (Group: glaucoma, control) mixed factorial ANOVA, with Location as within-subject factor and Group as between-subject factor. Also, for each stimulus pair, the total time dominance was initially analyzed with 3 (Percept: face, house/noise/spiral, mixed) × 3 (Location: central, LH, RH) × 2 (Group: glaucoma, control) mixed factorial ANOVAs, but in order to simplify the interpretation of the results we opted to report only planned ANOVAs that were of interest. For these, the Location and/or the Percept (i.e., face, house/noise patch/spiral, mixed) were the within-subject factors and Group was the between-subject factor. The ANOVA effects were adjusted with a Greenhouse-Geisser correction when violations of sphericity assumption were detected. In cases of multiple comparisons, the familywise error rate was controlled with the Bonferroni approach. Relationships among variables were examined with the Pearson product moment correlations. Alpha level was set at 0.05 for all tests.

## Results

### Rivalry rate

For the face/house pair, the 3 (Location: central, LH, RH) × 2 (Group: glaucoma, control) mixed factorial ANOVA revealed a significant Location × Group interaction effect *F*_(2, 52)_ = 3.6, *p* = 0.03, observed effect size η^2^= 0.04, while the Location main effect and the Group effect were not significant. Follow-up analysis after a significant interaction showed that rivalry rate of the glaucoma group was significantly lower than that of the control group, but only in the LH location. Moreover, for the control group only, rivalry rate in the central location was significantly lower than in the LH and RH locations. The results are shown in Figure 2A.

For the synthetic face/noise patch pair, the ANOVA revealed no significant main or interaction effects. For the central and RH locations, the rivalry rates were nearly identical for both groups. For the LH location, the rivalry rate was 16 ± 7 switches/min for the control group and 11 ± 6 switches/min for the glaucoma group, however this difference was not statistically significant. The results are shown in Figure 2B. For the synthetic face/spiral pair, the ANOVA revealed only a significant Group effect, *F*_(1, 26)_ = 5.8, *p* = 0.02, observed effect size η^2^= 0.15, while the Location main effect and Location × Group interaction were not significant. That is, the control group had significantly higher rivalry rates than the glaucoma group, at all stimulus locations. The rivalry rates were remarkably consistent for the 3 locations, as shown in Figure 2C. The mean and standard deviations of the rivalry rates for the 3 stimulus pairs are presented in Table 2.

**Table 2:** Means ± standard deviations of the rivalry rates for the control and glaucoma groups, for the three stimulus pairs and the three stimulus locations.

	**Control**			**Glaucoma**		
	**Central**	**LH**	**RH**	**Central**	**LH**	**RH**
Face/house	12 ± 4	15 ± 5	14 ± 4	12 ± 6	11 ± 4	13 ± 4
Synthetic face/noise patch	14 ± 4	16 ± 7	14 ± 5	13 ± 7	11 ± 6	13 ± 5
Synthetic face/spiral	16 ± 7	17 ± 8	16 ± 5	12 ± 6	12 ± 5	11 ± 5

Rivalry rates are reported in switches/min.

### Total time of exclusive percept dominance

**Figure 3:**
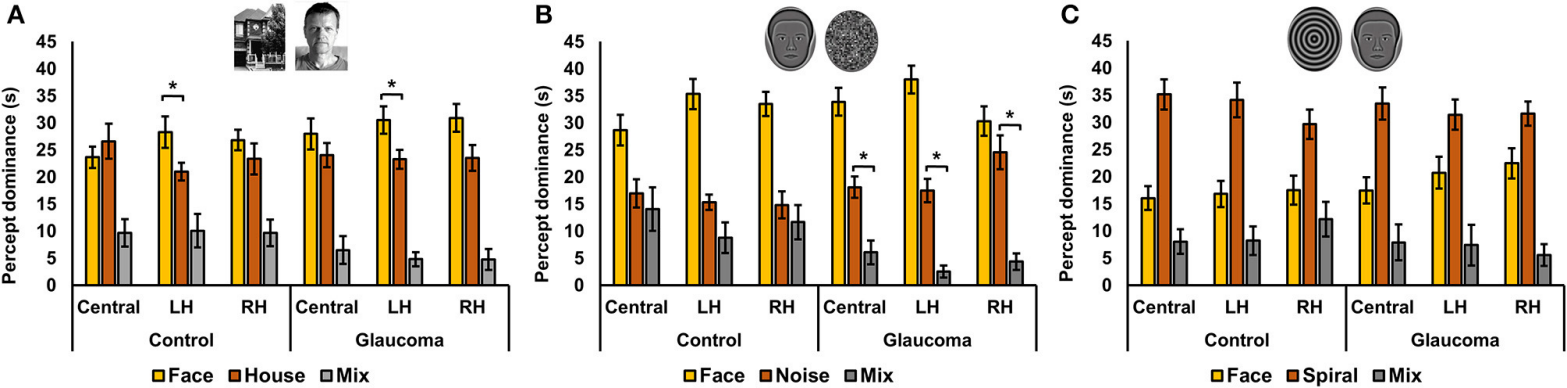
Total time of exclusive dominance at the three locations for the control and glaucoma groups, for the three stimulus pairs. **(A)** Shows the results for the face/house pair, **(B)** for the synthetic face/noise patch pair, and **(C)** for synthetic face/spiral pair. Error bars are ±1 SE. **(B)** Shows that the synthetic face dominated more than the noise patch in all conditions, while **(C)** shows that the spiral dominated more than the synthetic face in all conditions. The star sign * highlights other most relevant significant results.

For the face/house stimulus pair, we first examined the total time dominance for the mixed percept with a 3 (Location: central, LH, RH) × 2 (Group: glaucoma, control) mixed factorial ANOVA. No significant main effects or interaction were found. That is, the total time dominance of the mixed percept did not differ for the 2 groups and for the 3 stimulus locations. Time of exclusive dominance of the face and the house percepts were analyzed separately for each stimulus location with separate 2 (Percept: face, house) × 2 (Group: control, glaucoma) mixed factorial ANOVAs. For the central and RH locations, the analyses revealed no significant differences: the time dominance of the face percept was similar to that of the house percept, for both groups. For the LH location, the Percept main effect was significant, *F*_(1, 26)_ = 7.3, *p* = 0.01, observed effect size η^2^= 0.17, but there was no interaction or Group effect. Overall, in the LH location the time of exclusive dominance was longer for the face than for the house, for both groups. The results are shown in Figure 3A.

For the synthetic face/noise patch pair, a clear face dominance was observed, as shown in Figure 3B. We performed several planned ANOVAs that were of interest. First, we examined the total time dominance for the mixed percept with a 3 (Location: central, LH, RH) × 2 (Group: glaucoma, control) mixed factorial ANOVA. The analysis revealed a significant Location main effect, *F*_(2, 52)_ = 3.46, *p* = 0.039, observed effect size η^2^ = 0.034 and a significant Group effect, *F*_(1, 26)_ = 4.9, *p* = 0.035, observed effect size η^2^ = 0.12, but no interaction effect. Overall, the total time dominance of the mixed percept was the lowest for the LH location, although it was statistically significant only from that for the central location (*p* = 0.02). In addition, the time dominance of the mixed percept was significantly higher for the control group than for the glaucoma group.

Second, for the control group it was apparent that the mixed percept and the noise patch had similar time dominance. This was confirmed with a 3 (Location: Central, LH, RH) × 3 (Percept: face, noise, mixed) repeated measures ANOVA. The analysis revealed a significant Percept effect, *F*_(2, 26)_ = 15.9, *p* < 0.001, observed effect size η^2^ = 0.44, but this was driven by the large magnitude of the face time dominance; the mixed and noise dominated similarly at all stimulus locations. The same analysis revealed that this was not the case for glaucoma group. In this group, the Percept effect was also significant *F*_(2, 26)_ = 62.6, *p* < 0.001, observed effect size η^2^ = 0.66, but the follow up analysis showed that the time dominance for the face, noise and mixed percept were all highly significantly different one from another (*p* < 0.001).

For the synthetic face/spiral pair, the results presented in Figure 3C suggested the spiral dominated longer than the face for both groups and stimulus locations, and that the pattern of time dominance for the face, spiral and mixed percepts were similar across groups and locations. Indeed, separate 3 (Percept: face, spiral, mixed) × 2 (Group: control glaucoma) mixed factorial ANOVAs revealed the same pattern of result for all 3 stimulus locations: there was no difference between the 2 groups, but there was a highly significant Percept effect, lowest *F*_(2, 52)_ = 23.3, *p* < 0.001, observed effect size η^2^ = 0.47, for the RH analysis. Follow-up analyses indicated that the overall spiral dominated longer than the face, while the face dominated longer than the mixed percept (highest *p* = 0.002).

### Relationships between rivalry rates and clinical measures

We examined the relationships between clinical measures of the glaucoma group and the rivalry rates at the LH location for the face/house and synthetic face/noise patch pairs, and rivalry rates for the synthetic face/spiral pair, averaged for the 3 locations. The rivalry rates at these locations were of interest because they were the ones significantly different from those of the controls. Clinical measures were functional (i.e., visual acuity, stereo acuity, and visual field sensitivity) and structural measures (i.e., RNFL thickness, average and vertical cup-to-disc ratio, and IOP). Similar to our previous reports, no relationships between rivalry rates and clinical measures were found. Likewise, no relationships between the rivalry rates at these locations and visual acuity and stereo acuity were detected in the control group.

### Relationships between rivalry rates for different stimulus pairs and locations, for the 2 groups

The relationships among rivalry rates for the 3 stimulus pairs were examined for the 2 groups separately at each location. That is, for each group, we examined the relationships among rivalry rates of the face/house, synthetic face/noise patch, and synthetic face/spiral stimulus pairs for the central location, then for the LH location, and finally for the RH location. Interestingly, for both groups, rivalry rates of the 3 stimulus pairs correlated highly with each other for the central as well as for the RH locations, but this was not the case for the LH location. In this location, no significant correlation was found. The correlation coefficients are shown in Table 3.

**Table 3:** Correlation coefficients for the relationships among rivalry rates for the 3 stimulus pairs, at the central, LH, and RH locations, for the control and glaucoma groups.

	**Control**			**Glaucoma**		
	**Central**	**LH**	**RH**	**Central**	**LH**	**RH**
Face/house vs. synthetic face/noise	0.71^**^	0.26	0.78^**^	0.69^*^	0.42	0.60^*^
Face/house vs. synthetic face/spiral	0.61^*^	0.43	0.71^**^	0.64^*^	0.09	0.64^*^
Synthetic face/noise vs. face/spiral	0.32	0.45	0.65^*^	0.65^*^	−0.11	0.58^*^

^*^p < 0.05; ^**^p < 0.01.

## Discussion

This study used the binocular rivalry phenomenon to probe the function of stimulus-specific cortical areas involved in face perception in patients with early glaucoma. Three stimulus pairs of different complexity were used: in all, one stimulus was a face (real or synthetic) and the other a different visual object (i.e., house, noise patch, spiral). Depending on the complexity of the stimulus pair, different patterns of results were obtained. The main findings of this study are: (1) for the complex global stimulus pair real face/house, the glaucoma group had lower rivalry rate than the control group in the LH location; (2) a similar pattern of results but with a diminished magnitude was observed when the face stimulus had reduced complexity (i.e., synthetic face) and the other was a simple stimulus with no global features (i.e., a noise patch); (3) when both stimuli were global with reduced complexity (i.e., synthetic face/spiral pair), the glaucoma group had lower rivalry rate than the control group at all stimulus locations, a pattern of result that we previously reported for grouping during binocular rivalry using traditional orthogonal sinewave gratings (Issashar Leibovitzh et al., 2022). Taken together, these results indicate that the glaucoma group had atypical rivalry responses to faces during binocular rivalry and reveal a different pattern of responses for complex rivalry stimuli than for simple ones, which is in accordance with Wilson's hierarchical model of binocular rivalry (Wilson, 2003).

The main purpose of this study was to examine binocular rivalry responses in patients with early glaucoma using complex stimuli that are known to be processed in stimulus specific areas to probe the function of these neural structures. For this, we used a face and a house, both broadband in spatial frequency and orientation, and matched in contrast. In a landmark study, Tong et al. (1998) showed that visual perception of the face and the house during binocular rivalry matched the activity of the FFA and the PPA, respectively. However, this was also true for a replay of the responses condition, in which no rivalry was involved, suggesting that rivalry processes are already resolved earlier in the visual system, although it is not yet known where exactly this takes place. Nevertheless, we report that the glaucoma group had lower rivalry rates than healthy controls, but only when the stimuli were presented in the LH. Also, in this hemifield, the face dominated significantly longer than the house in both groups, while in the central and the RH, the face and the house time dominance were equal. What could possibly contribute to the lateralization and this pattern of responses that is so radically different from binocular rivalry with traditional stimuli in patients with glaucoma (Tarita-Nistor et al., 2019, 2020; João et al., 2021; Issashar Leibovitzh et al., 2022)? Our tentative answer to this question is as follows:

The most recent understanding of face processing involves a distributed neural network divided into a core system and an extended system (Haxby et al., 2000; Hildesheim et al., 2020). The core system is composed of (1) OFA in the inferior occipital gyrus which is involved in the initial representation of faces, (2) the FFA in the mid-fusiform gyrus in the inferior temporal cortex which is involved in face identification, and (3) posterior superior temporal sulcus (pSTS) which is concerned with the dynamic changes of faces such as gaze or mouth changes and facial expressions. The extended system is composed of non-face-specific regions such as the inferior frontal gyrus, the orbitofrontal cortex, the amygdalae and the insula that are task-specific and are activated when additional information is extracted during face processing, such as occurs with emotion, attractiveness, or biographical information. The neural structures of the core system in the occipito-temporal cortex are interconnected and typically right (hemispheric)-lateralized (Haxby et al., 2000; Hildesheim et al., 2020). Given that the faces used as the stimuli in this study were static and neutral, only the OFA and the FFA in the core system were likely involved in the face processing.

The right (hemispheric)-lateralization of the neural structures in the core system for face perception has been well-documented (Haxby et al., 2000; Pitcher et al., 2007; Yovel et al., 2008; Frässle et al., 2016; Hildesheim et al., 2020), but there is no lateralization for PPA, the stimulus-specific area that processes objects such as houses (Tong et al., 1998). This may explain the advantage of face dominance over house dominance in the LH (right hemisphere) for both groups. Also, in glaucoma, neuroimaging studies have shown changes in many neural structures throughout the brain, but interestingly, the disease seems to selectively affect the right hemisphere in areas where the structures of the core system are found, including right inferior occipital gyrus, right inferior temporal gyrus, right occipital lobe white matter, as well as the inferior longitudinal fasciculus (Chen et al., 2013; Williams et al., 2013; Nucci et al., 2020). It seems plausible that neural changes in the structures involved in early face-processing stage (specifically, OFA and FFA) and their reciprocal connectivity may affect rivalry responses when stimuli are presented to the LH (i.e., right hemisphere) only. The lower rivalry rate in the LH location of the glaucoma group may be due to strong reciprocal inhibitory interactions between populations of neurons and/or longer neural adaptation of the activity associated the dominant stimulus during rivalry. Alas, while it is not known where the rivalry processing takes place for these higher order visual stimuli, we can possibly conclude that the differences in rivalry rate in the LH matches the imaging reports showing anomalies in areas involved in face perception in the right hemisphere of patients with glaucoma.

Interestingly, we found that for control participants rivalry rate of the complex stimulus pair (i.e., real face/house) was significantly lower in the central location than in the LH and RH locations. This result is counterintuitive because it has been shown that the rivalry rate of orthogonal sinewave gratings is lower when stimuli are presented in eccentricity than centrally (Tarita-Nistor et al., 2020). However, these findings may not apply to complex stimuli in accordance with the 2 stages of the rivalry processing model proposed by Wilson (2003). It is difficult to fully explain these results because it is not yet known where exactly the rivalry processes take place for the stimuli involving a face or a house; as shown above, the current evidence from the literature only points to the fact that rivalry is already resolved by the time the signals reach FFA and PPA (Tong et al., 1998). Nevertheless, we believe that because the face is predominantly processed in the right hemisphere while the house in both hemispheres, there is a need of information transfer and synchronization of rivalry processes when the stimuli are presented to the RH (i.e., left hemisphere) and centrally, respectively. This is not the case when these stimuli are presented to the LH (i.e., right hemisphere). Specifically, for the RH condition (i.e., left hemisphere), there is information transfer required to process the face in the right hemisphere, but this does not apply to the house. Therefore, the rivalry processes for this condition may be between a stimulus in the right hemisphere (i.e., the face) and one in the left hemisphere (i.e., the house), which may not affect the rivalry rate, rendering it similar to the rivalry rate within the right hemisphere for the LH condition. For the central location, the input reaches both hemispheres, but the face is processed primarily in the right hemisphere, while the house in both. This means that for the central condition, the face can rival with the house in the right hemisphere (within hemisphere) and with the house in the left hemisphere (between hemispheres). These processes need to be synchronized and this may delay the perceptual switching. In fact, this condition is the only one where the house dominates slightly longer than the face (i.e., average of 27s vs 24s), albeit not statistically significant.

We also explored rivalry dynamics of the synthetic face/noise patch stimulus pair. The synthetic face has reduced complexity and represents the basic features of a neutral face, but it is still a global stimulus that likely engages feedback from the extrastriate cortex to the V1 for spatial organization of a large population of neurons involved in early stage of visual processing. The noise patch is a simple stimulus without a coherent spatial structure, and therefore is not global (Alais and Melcher, 2007). For the synthetic face/noise patch pair, the rivalry rate pattern was strikingly similar to that observed for the face/house pair, although for the LH location the results failed to reach significance. For both the glaucoma and control groups, the synthetic face dominated longer than the noise patch, suggesting a higher depth of suppression of the face over the noise patch. This outcome is consistent with other reports showing that during binocular rivalry, a face dominates more than a pattern stimulus (Yu and Blake, 1992). Also, the control group reported significantly higher mixed percept; for controls, the time dominance of the mixed percept was the same as that of the noise patch. It has been shown that in healthy controls, a complex stimulus such as a face rivals globally only when the fellow stimulus is also global, but this was not the case with our stimulus pair (Alais and Melcher, 2007). However, it appears that the visual system of patients with glaucoma treated the noise patch as a global stimulus because the time dominance of the mixed percept was minimal. This suggests stronger organizing effects of global feedback from extrastriate cortex to the V1 in order to allow for a coherent image formation of a non-global stimulus.

When both rivalry stimuli were global, but with reduced complexity (i.e., synthetic face/spiral stimulus pair), the glaucoma group had a lower rivalry rate than the control group, at all 3 stimulus locations. This finding is consistent with our previous report on perceptual grouping during binocular rivalry using orthogonal sinewave gratings as stimuli (Issashar Leibovitzh et al., 2022). Perceptual grouping during rivalry occurs when 2 adjacent stimuli with similar features (for example, sinewave gratings with the same orientation and spatial frequency) are perceived at the same time, and may be mediated by lateral connections of the cortical hypercolumns in the visual cortex (Alais and Blake, 1999). It appears that binocular rivalry processes for the synthetic face/spiral stimulus pair are similar to that of the 2 low-level stimuli for which grouping occurs (Arnold et al., 2009). However, it is not clear whether the low rivalry rate in glaucoma is due to weaker lateral connectivity during grouping or due to deeper inhibitory processes during binocular rivalry.

Finally, we also report that rivalry rates of the 3 stimulus pairs correlated highly with each other in the central and RH locations, but no significant correlation was detected in the LH, for both groups. This also implies a lateralization of responses, involving different rivalry processes for the 3 stimulus pairs. Overall, our findings agree with the Wilson's hierarchical model of binocular rivalry which suggests that rivalry processes of complex stimuli may involve different mechanisms than those of simple stimuli that are handled earlier in the visual system (Wilson, 2003).

We acknowledge that this study has some limitations. First, visual field and OCT data were not collected for the healthy control participants because the safety concerns for COVID-19 exposure had restricted the access to the instruments for patients only. All healthy control confirmed that they had had a relatively recent ophthalmic examination (within 2 years) with no findings of any ocular pathologies, nor suspicion for pre-perimetric glaucoma, but a corroboration of their verbal report with visual field test and OCT scans performed in the clinic for this study would have been ideal. We found that the control group had higher rivalry rates than the glaucoma group for specific conditions. If control participants had pre-perimetric glaucoma, then it is likely that the rivalry rates would have been diminished for this group too and no differences between the groups would have been found. Nevertheless, we acknowledge that all these issues are pertinent to the results presented in this study and should be addressed in future research. Second, we used 3 stimulus pairs, but other or more stimulus pairs may have been beneficial. The 2 rivalrous stimuli presented in each of the 3 stimulus pairs were carefully matched in size and contrast: the face/house stimulus pair consisted of coherent global stimuli that were broadband in spatial frequency; the synthetic face/noise patch pair had one stimulus that was global but with reduced complexity (i.e., synthetic face) and one simple stimulus with no coherent spatial structure (i.e., noise patch); and the synthetic face/spiral pair consisted of 2 global stimuli with reduced complexity. It has been pointed to us that for the synthetic face/noise patch pair, a phase-scrambled synthetic face would have been a better choice than the noise patch because it matches the characteristics of the synthetic face better. We agree that this is an issue that should be explored in future studies. We believe that such a condition would probably confirm the results presented in the present study at least for the control group, because it has been shown that for subjects with healthy vision, rivalry coherence (i.e., cumulative exclusive dominance of either stimulus) for a real face/phase-scrambled face stimulus pair is very similar to those for a real face/gratings pair or a gratings/gratings pair (Alais and Melcher, 2007). Finally, it has been shown that visual performance for patients with glaucoma is worse than in controls for a wide range of luminances, and this may affect rivalry rate (Bierings et al., 2018). However, this effect may depend on the glaucoma severity and may not be as strong for patients with pre-perimetric glaucoma who are carefully screened for the absence of visual field defects such as ours. For example, Essock et al., found that patients with early glaucoma and controls do not differ in terms of critical flicker frequency, while Bierings et al., show significant impairment on this measure in patients with moderate to severe glaucoma (median visual field MD of −14.4 dB) (Essock et al., 1996; Bierings et al., 2018). Despite the fact that the patients included in our study did not have visual field defects, we cannot claim that they did not have any deficits in any range of luminance level. However, we believe that this was not an issue in our sample because such an impairment would have affected rivalry rates across all conditions, not only in specific conditions, for example only in the LH condition for the face/house stimulus pair. In future studies, a luminance threshold for which patients with pre-perimetric glaucoma and healthy controls have the same performance should be applied to each stimulus pair.

In conclusion, our study reports atypical responses to faces during binocular rivalry in patients with early glaucoma. The pattern of responses depends on the stimulus complexity. Interestingly, for the most complex stimulus pair (i.e., face/house), the results reveal a lateralization of responses that mirrors the laterality of the core system network involved in face perception (specifically, OFA and FFA). These results may suggest early neurodegenerative processes affecting these structures and their reciprocal connectivity starting in the initial stage of glaucoma.

## Data availability statement

The raw data supporting the conclusions of this article will be made available by the authors, without undue reservation.

## Ethics statement

The studies involving human participants were reviewed and approved by the University Health Network Research Ethics Board. The patients/participants provided their written informed consent to participate in this study.

## Author contributions

GI managed the project, participated in study design, performed patient recruitment, collected and interpreted the data, provided clinical expertise, and was a major contributor in writing the manuscript. GT provided resources, secured funding, participated in study design, interpreted data, provided clinical expertise, and was a contributor in writing the manuscript. IK provided resources and was a contributor in writing the manuscript. YB provided resources, participated in study design, and was a contributor in writing the manuscript. LT-N conceptualized the study, provided resources, secured funding, analyzed and interpreted the data, and was a major contributor in writing the manuscript. All authors contributed to the article and approved the submitted version.

## References

[B1] Alais D. Blake R. (1999). Grouping visual features during binocular rivalry. Vis. Res. 39, 4341–4353. 10.1016/S0042-6989(99)00146-7 10789428

[B2] Alais D. Melcher D. (2007). Strength and coherence of binocular rivalry depends on shared stimulus complexity. Vis. Res. 47, 269–279. 10.1016/j.visres.2006.09.003 17049579

[B3] Arnold D. H. James B. Roseboom W. (2009). Binocular rivalry: spreading dominance through complex images. J. Vis. 9, 1–9. 10.1167/9.13.4 20055537

[B4] Bierings R. A. J. M. de Boer M. H. Jansonius N. M. (2018). Visual performance as a function of luminance in glaucoma: the de vries-rose, Weber's, and Ferry-Porter's Law. Invest. Ophthalmol. Visual Sci. 59, 3416–3423. 10.1167/iovs.17-22497 30025071

[B5] Boucard C. C. Hanekamp S. Curčić-Blake B. Ida M. Yoshida M. Cornelissen F. W. (2016). Neurodegeneration beyond the primary visual pathways in a population with a high incidence of normal-pressure glaucoma. Ophthalmic Physiol. Opt. 36, 344–353. 10.1111/opo.12297 27112227

[B6] Carvalho J. Invernizzi A. Martins J. Jansonius N. M. Renken R. J. Cornelissen F. W.. (2021). Visual field reconstruction using fMRI-based techniques. Transl. Vis. Sci. Technol. 10, 25. 10.1167/tvst.10.1.25 33520421 PMC7814355

[B7] Carvalho J. Invernizzi A. Martins J. Renken R. J. Cornelissen F. W. (2022). Local neuroplasticity in adult glaucomatous visual cortex. Sci. Rep. 12, 21981. 10.1038/s41598-022-24709-1 36539453 PMC9767937

[B8] Chen W. W. Wang N. Cai S. Fang Z. Yu M. Wu Q.. (2013). Structural brain abnormalities in patients with primary open-angle glaucoma: a study with 3T MR imaging. Invest. Ophthalmol. Vis. Sci. 54, 545–554. 10.1167/iovs.12-9893 23258150

[B9] Essock E. A. Fechtner R. D. Zimmerman T. J. Krebs W. K. Nussdorf J. D. (1996). Binocular function in early glaucoma. J. Glaucoma 5, 395–405. 10.1097/00061198-199612000-00007 8946296

[B10] Frässle S. Paulus F. M. Krach S. Schweinberger S. R. Stephan K. E. Jansen A.. (2016). Mechanisms of hemispheric lateralization: asymmetric interhemispheric recruitment in the face perception network. Neuroimage 124(Pt A), 977–988. 10.1016/j.neuroimage.2015.09.055 26439515

[B11] Frezzotti P. Giorgio A. Motolese I. De Leucio A. Iester M. Motolese E.. (2014). Structural and functional brain changes beyond visual system in patients with advanced glaucoma. PLoS ONE. 9, e105931. 10.1371/journal.pone.0105931 25162716 PMC4146554

[B12] Garaci F. G. Bolacchi F. Cerulli A. Melis M. Spanò A. Cedrone C.. (2009). Optic nerve and optic radiation neurodegeneration in patients with glaucoma: *in vivo* analysis with 3-T diffusion-tensor MR imaging. Radiology 252, 496–501. 10.1148/radiol.2522081240 19435941

[B13] Gupta N. Ang L. C. de Noël Tilly L. Bidaisee L. Yücel Y. H. (2006). Human glaucoma and neural degeneration in intracranial optic nerve, lateral geniculate nucleus, and visual cortex. Br. J. Ophthalmol. 90, 674–678. 10.1136/bjo.2005.086769 16464969 PMC1860237

[B14] Gupta N. Yücel Y. H. (2007). Glaucoma as a neurodegenerative disease. Curr. Opin. Ophthalmol. 18, 110–114. 10.1097/ICU.0b013e3280895aea 17301611

[B15] Haxby J. V. Hoffman E. A. Gobbini M. I. (2000). The distributed human neural system for face perception. Trends Cogn. Sci. 4, 223–233. 10.1016/S1364-6613(00)01482-0 10827445

[B16] Hernowo A. T. Boucard C. C. Jansonius N. M. Hooymans J. M. Cornelissen F. W. (2011). Automated morphometry of the visual pathway in primary open-angle glaucoma. Invest. Ophthalmol. Vis. Sci. 52, 2758–2766. 10.1167/iovs.10-5682 21398286

[B17] Hildesheim F. E. Debus I. Kessler R. Thome I. Zimmermann K. M. Steinsträter O.. (2020). The trajectory of hemispheric lateralization in the core system of face processing: a cross-sectional functional magnetic resonance imaging pilot study. Front. Psychol. 11, 507199. 10.3389/fpsyg.2020.507199 33123034 PMC7566903

[B18] Issashar Leibovitzh G. Trope G. E. Buys Y. M. Tarita-Nistor L. (2022). Perceptual grouping during binocular rivalry in mild glaucoma. Front. Aging Neurosci. 14, 833150. 10.3389/fnagi.2022.833150 35693345 PMC9175031

[B19] João C. A. R. Scanferla L. Jansonius N. M. (2021). Binocular interactions in glaucoma patients with nonoverlapping visual field defects: contrast summation, rivalry, and phase combination. Invest. Ophthalmol. Vis. Sci. 62, 9. 10.1167/iovs.62.12.9 34505864 PMC8434749

[B20] Kass M. A. Heuer D. K. Higginbotham E. J. Johnson C. A. Keltner J. L. Miller J. P.. (2002). The ocular hypertension treatment study: a randomized trial determines that topical ocular hypotensive medication delays or prevents the onset of primary open-angle glaucoma. Arch. Ophthalmol. 120, 701–713. 10.1001/archopht.120.6.701 12049574

[B21] Kass M. A. Heuer D. K. Higginbotham E. J. Parrish R. K. Khanna C. L. Brandt J. D.. (2021). Ocular hypertension study group. assessment of cumulative incidence and severity of primary open-angle glaucoma among participants in the ocular hypertension treatment study after 20 years of follow-up. JAMA Ophthalmol. 139, 1–9. 10.1001/jamaophthalmol.2021.0341 33856434 PMC8050785

[B22] Lisboa R. Leite M. T. Zangwill L. M. Tafreshi A. Weinreb R. N. Medeiros F. A. (2012). Diagnosing preperimetric glaucoma with spectral domain optical coherence tomography. Ophthalmology 119, 2261–2269. 10.1016/j.ophtha.2012.06.009 22883689 PMC3787835

[B23] McCulloch D. L. Loffler G. Colquhoun K. Bruce N. Dutton G. N. Bach M.. (2011). The effects of visual degradation on face discrimination. Ophthalmic Physiol. Opt. 31, 240–8. 10.1111/j.1475-1313.2011.00828.x 21410744

[B24] Nucci C. Garaci F. Altobelli S. Di Ciò F. Martucci A. Aiello F.. (2020). Diffusional kurtosis imaging of white matter degeneration in glaucoma. J Clin Med. 9, 3122. 10.3390/jcm9103122 32992559 PMC7600134

[B25] Pitcher D. Walsh V. Yovel G. Duchaine B. (2007). TMS evidence for the involvement of the right occipital face area in early face processing. Curr. Biol. 17, 1568–1573. 10.1016/j.cub.2007.07.063 17764942

[B26] Quigley H. A. Broman A. T. (2006). The number of people with glaucoma worldwide in 2010 and 2020. Br. J. Ophthalmol. 90, 262–267. 10.1136/bjo.2005.081224 16488940 PMC1856963

[B27] Shiga Y. Aizawa N. Tsuda S. Yokoyama Y. Omodaka K. Kunikata H.. (2018). Preperimetric glaucoma prospective study (PPGPS): predicting visual field progression with basal optic nerve head blood flow in normotensive PPG eyes. Transl. Vis. Sci. Technol. 7, 11. 10.1167/tvst.7.1.11 29372113 PMC5782826

[B28] Tarita-Nistor L. Samet S. Trope G. E. González E. G. (2019). Dominance wave propagation during binocular rivalry in mild glaucoma. Vis. Res. 165, 64–71. 10.1016/j.visres.2019.10.006 31678616

[B29] Tarita-Nistor L. Samet S. Trope G. E. González E. G. (2020). Intra- and inter-hemispheric processing during binocular rivalry in mild glaucoma. PLoS ONE 15, e0229168. 10.1371/journal.pone.0229168 32097443 PMC7041812

[B30] Tellouck L. Durieux M. Coupé P. Cougnard-Grégoire A. Tellouck J. Tourdias T.. (2016). Optic radiations microstructural changes in glaucoma and association with severity: a study using 3tesla-magnetic resonance diffusion tensor imaging. Invest. Ophthalmol. Vis. Sci. 57, 6539–6547. 10.1167/iovs.16-19838 27918827

[B31] Tham Y. C. Li X. Wong T. Y. Quigley H. A. Aung T. Cheng C. Y.. (2014). Global prevalence of glaucoma and projections of glaucoma burden through 2040: a systematic review and meta-analysis. Ophthalmology 121, 2081–2090. 10.1016/j.ophtha.2014.05.013 24974815

[B32] Tong F. Nakayama K. Vaughan J. T. Kanwisher N. (1998). Binocular rivalry and visual awareness in human extrastriate cortex. Neuron 21, 753–759. 10.1016/S0896-6273(00)80592-9 9808462

[B33] Varma R. Lee P. P. Goldberg I. Kotak S. (2011). An assessment of the health and economic burdens of glaucoma. Am. J. Ophthalmol. 152, 515–522. 10.1016/j.ajo.2011.06.004 21961848 PMC3206636

[B34] Williams A. L. Lackey J. Wizov S. S. Chia T. M. Gatla S. Moster M. L.. (2013). Evidence for widespread structural brain changes in glaucoma: a preliminary voxel-based MRI study. Invest. Ophthalmol. Vis. Sci. 54, 5880–5887. 10.1167/iovs.13-11776 23838767

[B35] Wilson H. R. (2003). Computational evidence for a rivalry hierarchy in vision. Proc. Natl. Acad. Sci. U. S. A. 100, 14499–14503. 10.1073/pnas.2333622100 14612564 PMC283620

[B36] Yovel G. Tambini A. Brandman T. (2008). The asymmetry of the fusiform face area is a stable individual characteristic that underlies the left-visual-field superiority for faces. Neuropsychologia 46, 3061–3068. 10.1016/j.neuropsychologia.2008.06.017 18639566

[B37] Yu K. Blake R. (1992). Do recognizable figures enjoy an advantage in binocular rivalry? J. Exp. Psychol. Hum. Percept. Perform. 18, 1158–1173. 10.1037/0096-1523.18.4.1158 1431750

[B38] Zhang N. Wang J. Li Y. Jiang B. (2021). Prevalence of primary open angle glaucoma in the last 20 years: a meta-analysis and systematic review. Sci. Rep. 11, 13762. 10.1038/s41598-021-92971-w 34215769 PMC8253788

[B39] Zhang Y. Q. Li J. Xu L. Zhang L. Wang Z. C. Yang H.. (2012). Anterior visual pathway assessment by magnetic resonance imaging in normal-pressure glaucoma. Acta Ophthalmol. 90, e295–302. 10.1111/j.1755-3768.2011.02346.x 22489916

